# Investigating mindfulness as the potential mediator between NBPA and wellbeing: an ecological dynamics interpretation

**DOI:** 10.3389/fpsyg.2025.1396095

**Published:** 2025-07-14

**Authors:** Tarek Michael Chouja, Eric Brymer, Luke Del Vecchio

**Affiliations:** Faculty of Health, Southern Cross University, Lismore, NSW, Australia

**Keywords:** nature based physical activity, green exercise, mindfulness, nature-based activities, psychological wellbeing, physical activity in nature

## Abstract

The wellbeing benefits of Nature Based Physical Activities (NBPA), also known as Green Exercise, are well documented. However, little is known about how the wellbeing benefits come about. Ecological dynamics (ED) theory suggests that NBPA facilitates mindfulness, which ultimately improves wellbeing. This scoping review systematically reviewed literature to examine how NBPA facilitates mindfulness, which contributes to wellbeing outcomes. It also evaluated the usefulness of the Ecological Dynamics (ED) framework in interpreting these findings. This review uniquely contributes to the emerging field of NBPA by applying the ED framework to understanding how NBPA might facilitate mindfulness in the emergence of wellbeing outcomes. Given the limited literature available, a scoping review was the preferred method. All searched articles were peer-reviewed, empirical articles, and full text in English published between Jan 2002 and Sep 2024. After an initial search of 1,571 articles across 14 databases, nine studies were included in the scoping review for complete analysis. Findings indicated that NBPA facilitates mindfulness and this connection produces wellbeing outcomes for individuals with little to no nature based or mindfulness experience. The review has highlighted a clear gap and opportunity in the emerging field of NBPA and mindfulness interventions for wellbeing outcomes for the general population and in environments other than natural forests like rural parks.

## 1 Introduction

This scoping review will examine how NBPA facilitates mindfulness, exploring the mechanisms through which different aspects of NBPA support the emergence of mindfulness and contribute to psychological well-being.

Recent research demonstrates that Nature based physical activity (NBPA), often referred to as green exercise, significantly enhances health and wellbeing beyond the impact of physical activity alone (Uher et al., 2021). However, there is little understanding of how these benefits are achieved. Traditional theories such as nature connectedness (NC), nature relatedness (NR), psycho-evolutionary theory (PET), and attention restoration theory (ART) do not fully explain how wellbeing is enhanced through NBPA (Araújo et al., 2019; Brymer et al., 2014; Connif and Craig, 2016; Manatti and Casado da Rocha, 2016; Van Heezik and Brymer, 2018). While these traditional theories have increased the understanding and importance of NBPA, critics have pointed out that these theories are limited because (1) they do not fully explain how physical activity in nature enhances wellbeing; (2) they fall short in developing a framework for encapsulating the complete and diverse range of human experiences in nature; and (3) they lack answers to the inherently complex and multi-dimensional aspect of the relationship between nature based physical activity and the environment (Araújo et al., 2019; Brymer et al., 2014; Connif and Craig, 2016; Manatti and Casado da Rocha, 2016; Van Heezik and Brymer, 2018).

Emergent studies suggest that NBPA might enhance wellbeing as it facilitates mindfulness (Araújo et al., 2019; Cervinka et al., 2020; Wolsko and Lindberg, 2013) which is supported by an ecological theoretical framework. Understanding how NBPA supports wellbeing is essential to support effective intervention designs for the benefit of individuals and communities.

Nature-based physical activity takes place in a variety of natural contexts, including green and blue spaces (Astell-Burt and Feng, 2021) and encompasses light to moderate physical activities such as trail walking, gardening, and adventure activities as well as blue space (water) activities like outdoor swimming, surfing, and water skiing (Wolsko and Lindberg, 2013; Coventry et al., 2021). The natural environments that facilitate NBPA range from diverse forests to urban parks. In addition to NBPA, these environments support a unique sensory stimulus, such as the coloring of the forest, the sounds of different species, the feel of the water or air on the skin and the clean air of the forest to bring about wellbeing outcomes (Cervinka et al., 2020; Wolsko and Lindberg, 2013; Coventry et al., 2021; Nisbet et al., 2019).

Previous research shows a positive link between NBPA and psychological wellbeing outcomes (Cervinka et al., 2020). The well-documented health benefits of engaging with natural environments include enhanced wellbeing for a diverse range of participants, regardless of the specific type of natural setting (Araújo et al., 2019; Braubach et al., 2021). These include: (1) benefits to short and long term mental health outcomes, (2) beneficial effects on perceived stress, (3) restorative health outcomes, and (4) quality of life and wellbeing outcomes (Cervinka et al., 2020; Braubach et al., 2021).

Wellbeing comprises the psychological, social, and physical capacities a person needs to flourish in daily life (Wassell and Dodge, 2015). Wellbeing has been described as fundamental to healthy living and not merely the absence of illness (Braubach et al., 2021). Psychological wellbeing is linked to an increase in spatial awareness (Nisbet et al., 2019) increased vitality, flourishing, and positive emotions (Wolsko and Lindberg, 2013), enhanced mental wellbeing such as a greater sense of perspective (Brymer et al., 2021); and an increase in a positive mood and decrease in perceived stress (Cervinka et al., 2020). Previously, mindfulness-based intervention strategies (MBIS) have been shown to improve mood and anxiety disorders and enhance wellbeing (Bajaj and Pande, 2016; Chiesa, 2013).

The notion of mindfulness can be traced to the ancient tradition of Buddhism (Anālayo, 2019). The modern approach to mindfulness has led to conceptualized frameworks focusing on State and Trait mindfulness (Davidson, 2010; Medvedev et al., 2017)^.^ Traditional perspectives on mindfulness, which focus on state qualities, refer to temporary states of mindfulness, such as being present in the moment. These elements of mindfulness include: (1) present moment awareness, (2) letting go of distracting thoughts, (3) being non-judgmental to the present moment, (4) equanimity, which is the self-regulation of unhelpful to helpful thoughts, emotions or sensations, (5) being immersed in the immediate environment by paying attention to the breath and the five senses of the body (sight, sound, touch, smell and taste) (Kabat-Zinn, 2005; Roychowdhury, 2021; Williams and Kabat-Zinn, 2013).

Recent research indicates that NBPA experiences, such as a guided walking tour of a forest naturally and effortlessly facilitate mindfulness (Araújo et al., 2019; Cervinka et al., 2020). If one of the critical attributes of mindfulness is immersion in the present moment, then the natural environment may support a mental state grounded in the present moment due to its rich, striking, and complete sensory stimulation (Brymer et al., 2021). Conceptually, natural environments invite an embodied awareness of the outer environment which in turn, facilitates an inner awareness that cultivates elements of mindfulness such as present moment awareness (Araújo et al., 2019; Brymer et al., 2021). Given the right environmental and activity conditions, mindfulness benefits may arise during NBPA.

Recent Research (Brymer et al., 2021) further elucidates how NBPA facilitates mindfulness, particularly in enhancing both physical and psychological wellbeing. For instance, Brymer et al. (2021) suggested that functional interactions with nature, involving agency in body positioning and movement, facilitate greater levels of mindfulness. Furthermore, the sensory-rich landscape offers a mental break from overthinking, facilitating a sensory-rich distraction and diverting attention to more intense experiences in nature (Brymer et al., 2021). This evidence implies that mindfulness opportunities emerge effortlessly in natural environments. The mediating role of mindfulness serves as a foundation for exploring how NBPA supports wellbeing outcomes.

**Figure 1:**
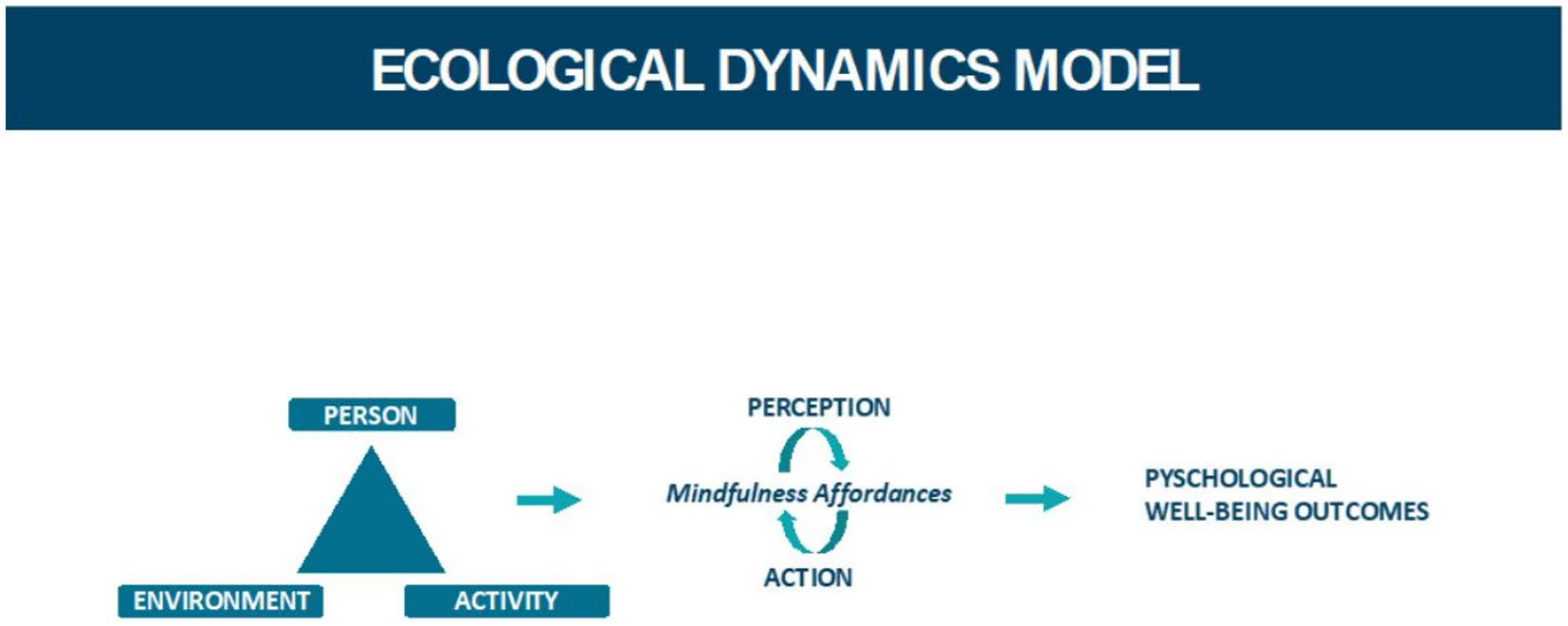
Ecological dynamics model – a conceptual model illustrating the interplay between green exercise and mindfulness according to the ecological dynamics’ framework.

Ecological Dynamics (ED) is a theoretical framework that combines key concepts from ecological psychology and dynamical systems theory to conceptualize behavior (see Figure 1). According to ED, behavior stems from the person-environment relationship (Araujo et al., 2020; Peacock et al., 2017). ED is grounded in an understanding that human beings are a part of the environment. The sensory rich quality of person-environment relationship facilitated by NBPA make it well-suited for mindfulness affordances (Brymer et al., 2014). Two essential concepts within ED are the notions of affordances and constraints.

The notion of affordances is a theory that has its roots in ecological psychology (Parker and Gibson, 1979) and refers to how the sensory rich environment, invites a set of behaviors where the person perceives the environment as affording a set of opportunities to walk or climb the terrain; or stop and absorb the sights and sounds of the environment (Parker and Gibson, 1979). In Ecological Dynamics, affordances are ever present in the person-environment relationship providing opportunities for behaviors. If the trail walk for instance is too difficult for that person to walk, the opportunities to interact in the environment are diminished (Withagen et al., 2012).

In previous years theorists have proposed that affordances are not merely opportunities for action, they are invitations for behaviors which can be accepted or rejected (Withagen et al., 2012). The environment is no longer considered as separate and instead invites a relationship where the person participates more actively in terms of searching for and actioning affordances such as climbing a rock or surveying the landscape for shelter or figuring out an alternative walking route (Brymer et al., 2014). From this perceptive nature might afford mindfulness experiences such as being present in the moment and being more connected to the environment (Brymer et al., 2021).

The notion of constraints describes the characteristics of the environment, individual and task performed that shape behaviors and actions during NBPA’s (Brymer et al., 2014). As explained in the ED framework, individual constraints include the sense of connection to nature, fitness skill and mindfulness skill. Environment constraints, include a challenging or easier forest path, the accessibility of the forest path and the bio diversity of the environment as being limiting or enabling. These are the necessary requirements imposed upon by the environment to undertake a forest walk. Equally, the requirements of the activity might vary based on several factors. These include the type of ‘task’ chosen (for example, walking a forest trail), the duration of the trail, and the regulation focus. The latter could be determined by how competitive the trail is, or whether it is simply for recreational purpose. The interaction of these constraints is crucial to the degree of mindfulness affordances that arise from the uniqueness of a sensory rich environment in influencing physical and psychological challenges upon the individual’s ability to perform a task such as trail walking (Brymer et al., 2014).

By considering the person-environment relationship from which wellbeing outcomes emerge, ED provides a useful framework for designing research on NBPA and mindfulness interventions. This approach ultimately contributes to a more comprehensive understanding of how these factors interact to promote overall wellbeing (Brymer et al., 2014).

Nature-based physical activity (NBPA) and mindfulness are two areas that have seen a surge of interest in recent years. Despite this, a significant gap in the literature persists: there is a lack of comprehensive understanding of how these two domains interact and potentially enhance each other. While existing studies have independently highlighted the benefits of mindfulness and NBPA, the connection between these two, that is how NBPA facilitates mindfulness for wellbeing outcomes remains underexplored, particularly through the lens of ecological dynamics.

This scoping review will examine how NBPA facilitates mindfulness, exploring the mechanisms through which different aspects of NBPA support the emergence of mindfulness and contribute to psychological well-being.

In doing so, this scoping review seeks not only to contribute to the theoretical understanding of the how NBPA facilitates mindfulness contributing to wellbeing outcomes, but also to offer practical insights for the development of NBPA interventions. It aims to provide a roadmap for researchers and practitioners alike, guiding the design of interventions that effectively leverage the NBPA for mindfulness leading to improved wellbeing outcomes.

## 2 Methods

### 2.1 Protocol

A scoping review was deemed the most appropriate method for this scoping review. Given that the research topic of how NBPA facilitates mindfulness contributing to wellbeing is relatively new, there are limited studies available. The review focused on examining how and if mindfulness emerges when performing NBPA. Through exploring this connection, the research also examined the emergence of wellbeing outcomes. Existing literature was assessed and examined through the perspective of the ED framework. We followed the established guidelines for scoping reviews as laid out in the PRISMA model (Page et al., 2021).

The scoping review protocol was registered with the Open Science Framework (OSF) and conducted according to the stages outlined by Arksey and O'Malley (2005). The following stages were adhered to: (i) Identifying the research question, (ii) Identifying relevant studies, (iii) scoping review selection, (iv) extracting the data, and (v) collating, summarizing, and reporting the results.

### 2.2 Stage one: Identifying the research question

The objective of this scoping review was to explore the available literature to determine how NBPA facilitates mindfulness in producing wellbeing outcomes. This included (1) determine through this connection if mindfulness emerges as a result of NBPA and following this (2) examine literature that met the criteria above to determine those that focused on wellbeing outcomes, (3) utilizing the Ecological dynamics framework to interpret the key findings of the scoping review. Four questions guided the review:

1. What is the special connection, if any, between NBPA and mindfulness?
2. Do NBPA inherently facilitate mindfulness?
3. Is the effectiveness of NBPA enhanced when mindfulness activities are included?
4. How does the interplay between NBPA and mindfulness contribute to wellbeing outcomes?

### 2.3 Stage two: Identifying relevant studies

A systematic search for primary research published between Jan 2002 and Sep 2024 was undertaken across electronic databases: Health source, Medline, CINAHL Plus, Psych and Behavioral Sciences selection, SPORT discus with Full text, ERIC, PubMed, ProQuest, Science Direct, Google Scholar, Education Complete, Academic Premier, APA Psych Info and Medline with Full text. Search terms were: “nature-based physical activity,” “green exercise,” “mindfulness,” “nature-based activity in nature” and “Psychological wellbeing.” The search strategy, including all identified keywords was adapted for each included database and screened for additional studies (refer to Table 1).

**Table 1:** Inclusion and exclusion criteria summary.

	Inclusion criteria	Exclusion criteria
Time period	2002–2024	Anything before 2002
Demographics	9 to 18 + years of age	Children under 9 years of age
Scoping review designs	peer reviewed journals; Primary research	Non-original research opinion pieces
Green exercise	Trail walks; water sports	Artificial or simulated environments
Mindfulness	Included elements of mindfulness outcomes	Studies that did not report mindfulness outcomes
Wellbeing Outcomes	Any type of wellbeing	None
Language	English	Studies not written in English
Databases	Health Source Medline; CINHL Plus; Psych and Behavioral Sciences; SPORT Discus; ERIC; Google Scholar; Pub Med; Pro Quest; Web of Sciences Medline with full text Education research complete Academic search premier Science direct APA PsycINFO	No source was excluded
Keywords	Green exercise; Mindfulness; Nature-based activities; Psychological wellbeing; Physical activity in nature	

The search strategy was conducted in consultation with an expert librarian to narrow the key search terms: green exercise or nature-based activity and mindfulness. All authors reviewed this to ensure the studies were filtered according to the key search terms (see Table 2).

**Table 2:** Database searches and results – key search terms across databases that did not produce citations relevant to the scoping review are not included in this table.

Databases and date range	Specific limits	Number of qualified citations imported to endnote
Health Source – Academic Edition Jan 2002–Sep 2024	Full text Peer reviewed Academic journal	47
Medline Jan 2002–Sep 2024	Full text Peer reviewed	209
CINAHL Plus Jan 2002–Sep 2024	Full text Peer reviewed	100
Psych and Behavioral Sciences Selection Jan 2002–Sep 2024	Full text Peer reviewed	40
SPORT Discus with Full Text Jan 2002–Sep 2024	Full text Peer reviewed	38
ERIC Jan 2002–Sep 2024	Full text Peer reviewed	27
Google Scholar Jan 2002–Sep 2024	Full text Peer reviewed	37
PubMed Jan 2002–Sep 2024	Full text Peer reviewed	3
ProQuest Jan 2002–Sep 2024	Full text Peer reviewed	214
Education complete Jan 2002–Sep 2024	Full text Peer reviewed	66
Medline with full text Jan 2002–Sep 2024	Full text Peer reviewed	330
Academic Premier Jan 2002–Sep 2024	Full text Peer reviewed	355
Science direct Jan 2002–Sep 2024	Full text Peer reviewed	69
APA PsycINFO Jan 2002–Sep 2024	Full text Peer reviewed	10,301

#### 2.3.1 Inclusion/exclusion criteria

Publications were included if they were published in peer-reviewed journals in English. All scoping review designs were included as long as the data focused on links to NBPA, mindfulness and wellbeing. The demographics of the scoping review designs included female and male participants ranging from children 9 years of age to adults.

Publications were excluded if they were not full text and did not report peer-reviewed primary research. Review papers were excluded from this scoping review because they are not original data, and the focus of this research was to evaluate primary data only. Articles published before Jan 2002 were excluded because the relevancy of the topic NBPA facilitating mindfulness contributing to wellbeing outcome is a recent phenomenon and therefore no studies, to our knowledge, existed prior to then.

Studies that did not include elements of mindfulness and NBPA were excluded as well as those that did not report any wellbeing outcomes in relation to mindfulness and NBPA (see Table 1).

### 2.4 Stage three: Scoping review selection

Following the systematic search through electronic databases, reference management software EndNote, (Version x9. 3.3., Clarivate Analytics Philadelphia, PA, United States) was used to import the relevant studies. After the removal of duplicates the authors independently screened the results by title and abstract using the Preferred Reporting Items for Systematic Reviews and Meta-Analysis (PRISMA) guidelines (Page et al., 2021).

A total of 1,571 studies were identified by electronic database and additional search criteria. The removal of duplicates, full-text retrieval and screening for inclusion criteria, left a total of nine studies that were included in this review.

**Figure 2:**
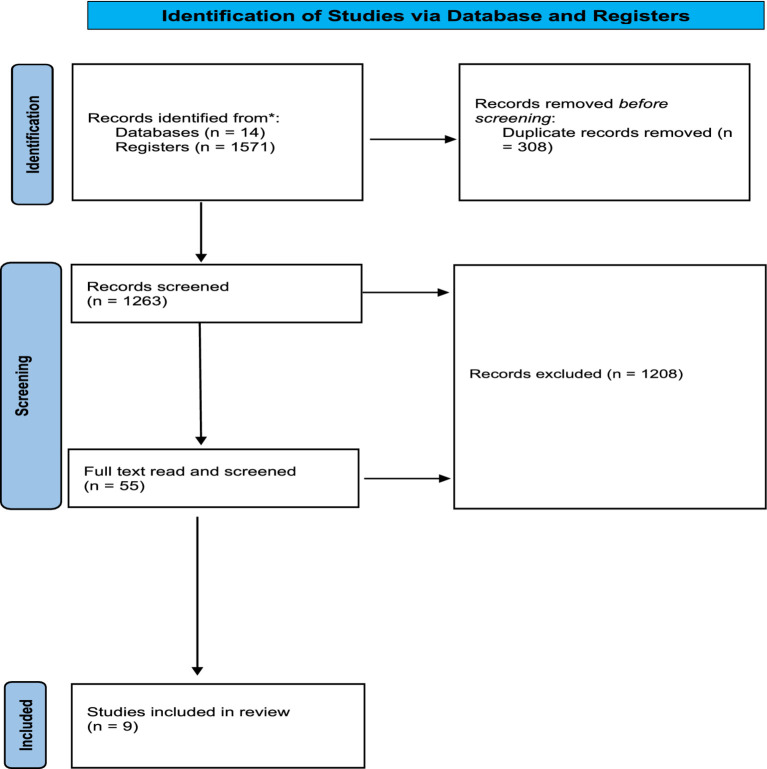
PRISMA flow diagram for new systematic reviews which included searches of databases and registers only.

Additionally, scoping reviews, systematic reviews and any other secondary research articles were excluded based on not being of primary research. The overview of our search process is depicted in the PRISMA flow diagram (see Figure 2) which specifies the selection of articles included in the review, removal of duplicates and the final screening.

### 2.5 Stage four: Extracting the data

Initial data extraction was conducted using a Microsoft Excel (Microsoft 2021) document, which chartered the five primary studies to be included in the final research. The key questions used to extract the precise data were (1) does NBPA facilitate mindfulness; (2) what this connection is; (3) how this connection comes about; (4) common themes that emerged from the studies. Chartering was then conducted by two independent reviewers to gauge the relevancy and accuracy of the studies relating to the scoping review’s aim and purpose.

### 2.6 Stage five: Collating, summarizing and reporting the results

Chartered scoping review results were imported into Microsoft Word (Microsoft 2021) and focused on key headings relating to the following: article characteristics (author, year and population), scoping review characteristics (Wellbeing outcomes and Type of NBPA) and the key findings evidencing how NBPA facilitates mindfulness. The data was reviewed by each author and, upon agreement, summarized in Table 3.

**Table 3:** Summary of results.

Author	Scoping review design	Population characteristics mean age/gender (years)	Sample characteristics clinical vs. non clinical and preventative vs. rehab	Types of NBPA	Frequency and duration	Outcome measures and methods used	Inter connection outcomes between NBPA and mindfulness
Brymer et al. (2020)	Qualitative Inductive and exploratory – in-depth interviews	Purposive sample *N* = 15 No age reported No gender reported	Non clinical Preventative focus	Nature-based recreation; Multiday treks; Short walks; Recreational visits to nature; Mountaineering Gardening	Not specified	Thematic analysis to determine perceptions of how nature-based experiences enhance mental wellbeing	All types of GE facilitate mindfulness through: (1) A sense of perspective. (2) Being immersed in the moment
Cervinka et al. (2020)	Cross sectional case scoping review: Quantitative questionnaires and physical experiment	*N* = 99 16–81 years old Mean Age 43.15 years, (SD 17.11) *F* = 64% M = 36%	Non clinical Preventative focus	Recreational forest tour consisting of walking 4 selected forest paths.	2.5-h trek	Mindfulness: Freiburg mindfulness inventory Mood states: PANAS Perceived stress: Single item stress score Connectedness: inclusion of nature in self-scale (INS) Reflective thinking: Widening One’s Mind (WOM)	Walking with guidance along specific paths in recreational forests produced an increase in mindfulness by 7% pre and post visits
Barrable et al. (2021)	Quantitative: Pre and post questionnaires and physical experiment	*N* = 74 children 9–10 years old, SD = 9.51 F = 29 M = 33 No data on sex = 12	Non clinical Preventative focus	Outdoor activities at a nature reserve including pretend hunting game	Activities ranged from 30 min to 2.5 h	Nature Connection: Nature connection index (NCI) Nature inclusion: Inclusion of nature in self (INS) Mood states: PANAS	Children’s experience of natural environments can be enhanced through mindfulness interventions
Wolsko and Lindberg (2013)	Quantitative survey based.	Scoping review 2 *N* = 223 (*M* = 33.30, SD = 12.86) F (61.4%) M (38.6%) Scoping review 3 *N* = 410 F = 60.7% M = 39.3%	Non clinical Preventative focus	Survey with selection of Outdoor activities	Frequency of activities determined by noting how many times a year the respondent reported undertaking green exercise activities	Mindfulness: Mindful Attention Awareness scale Connection with nature: CNS Psychological wellbeing: flourishing scale and SPANEOutdoor recreation patterns: self-reported Vitality: Subjective vitality scale	Connected to nature, mindfulness and outdoor recreation positively correlate to psychological wellbeing outcomes
	Quantitative Questionnaires	*N* = 100 Mean = 19.48 SD = 2.74 F = 58, M = 41 Unspecified = 1	Non clinical Preventative focus	Outdoor Walking in nature.	20 min	Trait mindfulness: MAAS State Mindfulness: TMS Mood states: PANAS Nature relatedness: NRS	Mindfulness meditation techniques facilitate a greater nature experience such as: Greater awareness of surroundings Stronger connectedness with nature
Yang et al. (2023)	Quantitative multi-site trial comparing NBT and control groups. Pre and Post-tests assessments	*N* = 291 *M* = 53.48 SD = 24.05 F = 225 M = 66	Mix of clinical and non-clinical Mix of preventative and rehab	Therapeutic Gardening	30 sessions For individuals not in the control group & no activity for individuals within the CG.	K-PSS for stress, K-SWLS for life satisfaction, and K-MAAS for mindfulness K-SWLS for life satisfaction MHS: A for anxiety symptoms	Significant improvements in mindfulness among participants in the gardening group, contributing to improvements in overall wellbeing.
Schaller and Karing (2024)	Quantitative Random control trial Pre and post interventions	*N* = 89 *M* = 23 SD = 4.36 F = 74 M = 15	Non clinical Preventative focus	Mindfulness app Nature only Combination of nature and mindfulness	7 weeks mindfulness or nature-based program use 7 weeks post intervention	Mindfulness: FFM@ Life satisfaction: HSWBS Depression: PHQ-8 Anxiety: GAD-7 Stress: PSS-10	Nature only intervention improved mindfulness, life satisfaction and stress.
Kang et al. (2023)	Quantitative structural equation modeling, two-wave autoregressive cross-lagged model	*N* = 276 *M* = 54.99 SD = 23.25 F = 204 M = 72	Mix of clinical and non-clinical Mix of preventative and rehab	Gardening program	30 sessions Twice weekly for 15 weeks	K-PSS for stress, K-SWLS for life satisfaction, and K-MAAS for mindfulness	Higher mindfulness achieved at post-intervention stage contributing to improved life satisfaction
Ward-Smith et al. (2020)	Qualitative Semi structured interviews and focus groups	N children = 25 M children = 12.5 SD = 1.15 F = 13 M = 12 N adults = 12 M children = 21.5 SD = 2.02 F = 13 M = 12	Non clinical Preventative focus	Reflexive Journaling Outdoor education	5-day camp: 2-h day hike, 2-h night hike, tree planting, physical course	Reflexive journaling; CNS; participant observation;	Nature based activities facilitated, self-reflection, calm, creativity, heightened sense of self-awareness

## 3 Results

This scoping review aimed to review, analyze and synthesize the current body of knowledge that supported an evidence-based approach to how NBPA facilitates mindfulness, affording wellbeing outcomes. The scoping review identified nine studies that contribute to understanding how NBPA facilitates mindfulness and how such a connection may bring about wellbeing benefits to a diverse population type.

### 3.1 Population and scoping review design

Nine studies were included based on the criteria aligned with the research objectives. Across these studies, there were 1,212 participants, with a gender distribution of 883 females, 435 males, and 28 participants with unreported gender. Ages ranged from 9 to 81 years. Scoping review designs were varied, with five studies employing qualitative methods, including inductive and exploratory in-depth interviews and semi-structured focus groups, while four studies used quantitative designs, such as cross-sectional, structural equation modeling, and randomized control trials.

### 3.2 Activity and environment types

All nine studies incorporated various forms of NBPA. Two studies focused on walking interventions: (1) a 2.5-h guided forest walk along four distinct forest paths (Cervinka et al., 2020), and (2) a 20-min walk featuring three variations: indoor, outdoor, and outdoor with a mindfulness intervention (Nisbet et al., 2019). Other activities included a pretend hunting game for children, spanning from 30 min to 2.5 h (Uher et al., 2021); diverse outdoor activities, such as hiking, cross-country skiing, and non-motorized boating (Wolsko and Lindberg, 2013); and gardening programs conducted over multiple weeks (Kang et al., 2023; Yang et al., 2023). Additional activities included short walks, mountaineering, and tree planting (Brymer et al., 2020; Ward-Smith et al., 2020).

### 3.3 Environment and NBPA prescription

Across the nine studies, diverse green exercise environments, from structured outdoor camps to university walkways, were utilized to examine how NBPA settings facilitate mindfulness and enhance well-being. These studies combined pre-and post-intervention assessments to evaluate the environments impact on participants’ mindfulness.

In a scoping review by Cervinka et al. (2020), participants’ mindfulness levels increased after a 2.5-h guided forest walk with varied trails, assessed through the Widening One’s Mind (WOM) scale. Barrable et al. (2021) found that nature-focused role-playing activities, such as a pretend hunting game for children, encouraged mindful interaction with the environment, supporting engagement with sensory aspects of nature.

Nisbet et al. (2019) highlighted that outdoor walking, especially with mindfulness instruction, led to higher mindfulness and connectedness compared to indoor walking. Wolsko and Lindberg (2013) noted that participants engaging frequently in outdoor activities, including hiking and skiing, reported greater vitality and positive mood, linking these well-being benefits to regular nature exposure.

Gardening studies by Kang et al. (2023) and Yang et al. (2023) demonstrated that consistent, structured gardening sessions over several weeks in therapeutic settings significantly improved mindfulness, life satisfaction, and stress reduction. Likewise, Schaller and Karing (2024) found that a nature only intervention over seven weeks promoted mindfulness and reduced stress.

Finally, Ward-Smith et al. (2020) showed that structured outdoor camps incorporating NBPAs like hiking and tree planting created opportunities for mindfulness and self-reflection.

### 3.4 NBPA facilitating mindfulness

#### 3.4.1 Presence

Studies indicate that engaging in NBPA encourages individuals to be fully present in the moment, cultivating a state of mindfulness that is associated with improved psychological well-being. Brymer et al. (2020) reported that activities such as trekking and mountaineering allowed participants to experience an intensified sense of immersion in their surroundings, promoting a mindfulness-oriented state where participants could focus on the present rather than external stressors. Schaller and Karing’s (2024) randomized control trial further reinforced these findings, demonstrating that both mindfulness and nature-based interventions independently promoted mental well-being through present-moment awareness.

#### 3.4.2 Nature connection

Beyond present-moment awareness, NBPA facilitates a profound sense of connection to the natural environment, which has been shown to improve mood and overall mental health. Nisbet et al. (2019) found that simply being in nature during a structured 20-min walk heightened participants’ connection to their surroundings, resulting in improved mood and a more mindful state of awareness. Similarly, Wolsko and Lindberg (2013) observed that frequent engagement in outdoor activities such as hiking, kayaking, and skiing correlated with increased psychological flourishing, subjective vitality, and positive emotional states. This connection to nature not only enhances mindfulness but also provides a sense of psychological restoration, offering an emotional sanctuary from daily stressors. Kang et al. (2023) further substantiated these effects in a longitudinal gardening program, where participants reported significant gains in life satisfaction and stress reduction attributed to the mindful engagement and nature connection fostered through regular gardening activities.

Table 3 details the characteristics of all nine studies included in this scoping review, summarizing the scoping review designs, population characteristics, types of NBPA, frequency, duration, and key outcome measures.

Table 4 details the facets of mindfulness, the description and outcomes of each scoping review.

**Table 4:** Facets of mindfulness explored.

Author	Facet of mindfulness	Description	Outcomes
Brymer et al. (2020)	Present-Moment Awareness	Heightened awareness and focus on immediate experiences in nature.	Participants described being fully absorbed in the moment through sensory engagement and environmental interactions, such as observing nature and feeling its rhythms.
	Non-Judgmental Awareness	Observing surroundings and internal experiences without critique or judgment.	Nature was perceived as non-judgmental and accepting, providing a space for reflection without societal pressures.
	Embodied Awareness	Awareness centered on physical sensations and interactions with the natural environment.	Participants noted being more in their bodies and less in their minds, with nature facilitating this shift through sensory experiences like touch, sound, and sight.
	Gratitude and Awe	Emotional responses to the beauty and interconnectedness of nature.	Participants reported feelings of gratitude and awe, describing nature as inspiring a sense of humility and appreciation for its vastness and processes.
Cervinka et al. (2020)	Present-Moment Awareness	Awareness of immediate experiences, including sensations and surroundings.	Measured pre-and post-visit using the Freiburg Mindfulness Inventory (FMI), assessing participants’ attention to their current experiences.
	Non-Judgmental Awareness	Observing thoughts and perceptions without critique.	Encouraged during guided visits, where participants were instructed to explore forest sites without judgment.
	Sensory Engagement	Active engagement of the senses with the natural environment.	Participants were instructed to explore forest areas through all senses (e.g., walking, touching, observing).
	Reflective Thinking	Capacity for introspection, re-evaluating perspectives, and generating new ideas.	Explored through the Widen One’s Mind (WOM) scale, which measures reflection, perspective-shifting, and idea generation facilitated by forest settings.
	Interconnectedness	Sense of connection to the forest and nature.	Evaluated using a modified Inclusion of Nature in Self (INS) scale
Barrable et al. (2021)	Present-Moment Awareness	Attention focused on current experiences in nature.	Activities like mindful listening to natural sounds and observing nature closely (e.g., flowers and mountains).
	Non-Judgmental Awareness	Observing nature without evaluation or critique.	Children were encouraged to adopt an open and accepting approach during mindfulness-based activities.
	Interconnectedness	Developing a sense of connection to nature and its ecosystems.	Measured using tools like the Nature Connection Index (NCI) and the Inclusion of Nature in Self scale (INS).
	Emotional Regulation	Promoting positive emotional states and reducing negative emotions through engagement.	Observed as increases in Positive Affect (PA) and decreases in Negative Affect (NA) scores post-activity.
Wolsko and Lindberg (2013)	Present-Moment Awareness	The ability to remain fully engaged and attentive to one’s current environment and experiences.	Measured using the Mindful Attention Awareness Scale (MAAS), assessing the frequency of paying attention to present sensations and surroundings.
	Present-Moment Awareness	Observing thoughts, emotions, and experiences without critique or evaluation.	Implicit in MAAS, focusing on participants’ ability to accept experiences as they unfold.
	Self-Reflection	Engaging in thoughtful examination of one’s emotions and connection with nature.	The MAAS scale to measure the frequency of present-moment awareness and attention to experiences
	Interconnectedness	Feeling of unity and oneness with the natural world.	Assessed through the Connectedness to Nature Scale (CNS), linking mindfulness with a deeper ecological identity.
	Present-Moment Awareness	The ability to focus attention on the present moment, observing events as they unfold.	Guided mindfulness intervention instructed participants to focus on their steps, surroundings, and sensory inputs like sounds and colors.
	Non-Judgmental Awareness	Observing thoughts and surroundings without critique or evaluation.	Participants were instructed to observe nature without judging, enhancing open and objective awareness of the environment.
	Curiosity	A sense of interest and curiosity about experiences and surroundings.	Measured using the Toronto Mindfulness Scale (TMS), assessing participants’ curiosity during the mindfulness walk.
	Decentering	Viewing experiences with detachment and disidentification from thoughts.	Evaluated as part of TMS, particularly focusing on participants’ ability to distance themselves from their immediate thoughts and reactions.
	Interconnectedness	Feeling of unity and connection with nature and the environment.	Observed through increased state nature-relatedness scores (INS) after mindfulness interventions in outdoor environments.
Yang et al. (2023)	Present-Moment Awareness	Ability to focus on the current moment and sensory experiences.	Mindful Attention Awareness Scale (MAAS) measured how participants stayed attentive during nature-based activities.
	Emotional Regulation	Managing emotional responses effectively in a calming environment.	Reduced anxiety, depression, and stress were observed post-intervention through therapeutic gardening sessions.
	Non-Judgmental Awareness	Observing thoughts and emotions without critique.	Participants reported improved ability to remain non-critical of internal experiences, likely influenced by natural stimuli.
	Self-Reflection	Engaging in deeper introspection and understanding personal experiences.	Nature-based interventions facilitated reflective practices through structured activities like gardening and journaling.
	Interconnectedness	Fostering a sense of connection to the environment and social connections.	Participants reported enhanced life satisfaction and reduced loneliness, indicating improved connection with others and nature.
Schaller and Karing (2024)	Observing	The ability to notice or attend to internal and external experiences such as sensations, thoughts, and emotions.	Measured through participant responses on the FFMQ, e.g., noticing sensory details like wind or sunlight.
	Describing	The ability to label internal experiences with words.	Participants rated their ability to articulate their thoughts and feelings during mindfulness practice.
	Acting with awareness	Staying focused on the present activity and avoiding automatic, mindless behavior.	Assessed through participant reflection on their attentiveness during mindfulness exercises or nature walks.
	Non-Judging of Inner Experience	Refraining from evaluating thoughts and feelings as good or bad.	Evaluated through FFMQ items related to accepting personal experiences without judgment.
	Non-Reactivity to Inner Experience	Allowing thoughts and feelings to come and go without being carried away by them.	Participants rated their ability to let go of intrusive thoughts during mindfulness practice.
Kang et al. (2023)	Present-Moment Awareness	Focus on the current moment and surroundings.	Mindful Attention Awareness Scale (MAAS) was used to measure participants’ attention and mindfulness levels.
	Emotional Regulation	Managing emotional responses effectively.	Post-intervention improvements in anxiety and depression indicated enhanced emotional regulation abilities.
	Non-Judgmental Awareness	Observing and accepting thoughts and emotions without critique.	Participants reflected nonjudgmentally during therapeutic gardening activities, fostering acceptance.
	Self-Reflection	Engaging in introspection to understand personal experiences and emotions.	Gardening and structured group discussions encouraged participants to engage in reflective thinking.
	Interconnectedness	Feeling of connection to nature and others.	Gardening activities and social interactions promoted unity with nature and group cohesion.
Ward-Smith et al. (2020)	Present-Moment Awareness	The ability to focus on the present moment and surroundings.	Participants described being attentive to sensory details during nature-based activities, such as hiking.
	Non-Judgmental Awareness	Observing thoughts and feelings without judgment.	Nature activities encouraged reflection without imposing expectations, promoting self-acceptance.
	Self-Reflection	Examining one’s internal states and emotions.	Participants used quiet moments in nature to contemplate personal thoughts and gain clarity.
	Emotional Regulation	Managing stress and fostering positive emotional states.	Nature immersion reduced stress and promoted feelings of calmness, joy, and relaxation.
	Interconnectedness	Developing a sense of connection with nature and others.	Participants reported a heightened sense of unity with the environment and a deeper awareness of interdependence.

## 4 Discussion

**Figure 3:**
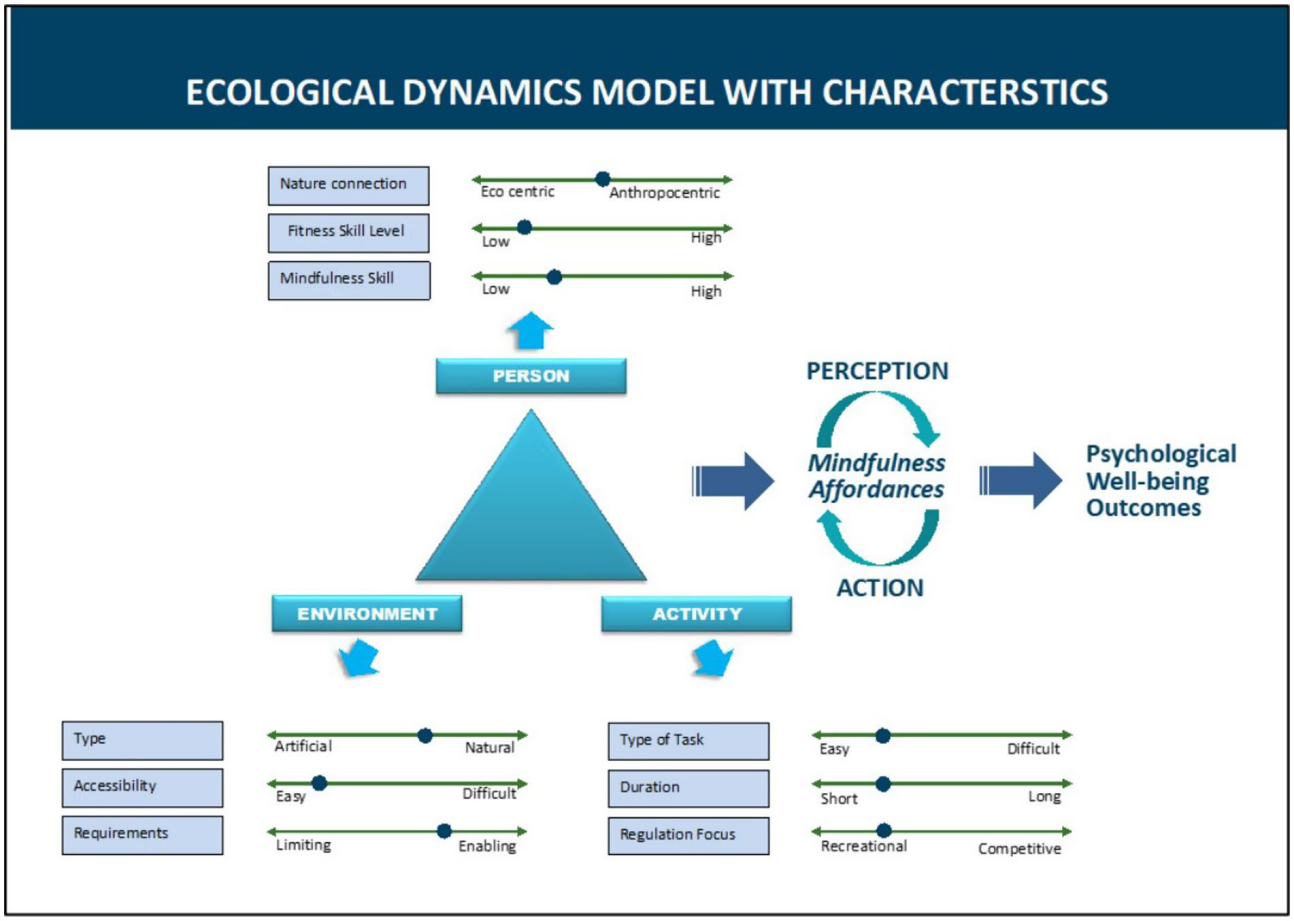
Ecological dynamics model with constraints – a conceptual model illustrating the interplay between green exercise and mindfulness according to the ecological dynamics framework. The added constraints display the essential relationship between the person, environment, and activity.

The scoping review set out to examine how NBPA facilitates mindfulness, exploring the mechanisms through which different aspects of NBPA support the emergence of mindfulness and contribute to psychological well-being. Four key questions were proposed to guide a response that would pave the way for understanding how NBPA facilitates mindfulness for wellbeing outcomes. The utilization of the modified ecological dynamics (ED) framework was included to elaborate on the key findings. This provides context to the results based on the triune relational components of: the person, the environment, and the activity (refer to Figure 3). The discussion will focus on the outcomes of the key findings as analyzed in the results section.

### 4.1 What is the relationship between green exercise and mindfulness?

The nine studies indicate a range of connections between NBPA and mindfulness. Several studies, including those with structured walking interventions, incorporated mindfulness techniques that participants could engage in without prior mindfulness experience. This suggests that natural settings inherently support mindful states by facilitating sensory immersion. Additionally, variations in activity frequency, duration, and environmental conditions (such as terrain difficulty) reveal a potential “dosage” effect of NBPA for mindfulness, pointing to a balanced approach that includes light to moderate physical engagement within accessible environments as more conducive to mindfulness.

This “dosage” effect highlights an opportunity to develop a more refined NBPA framework that includes specific guidance on activity types and natural settings, potentially enhancing access to mindfulness-promoting NBPAs across diverse population types and green spaces, including urban parks.

### 4.2 Does NBPA’s facilitate mindfulness?

Across the nine studies reviewed, findings indicate that NBPAs can effectively facilitate mindfulness, particularly when activities are immersive, sensory-rich, and not overly demanding (Brymer et al., 2021). The natural environment itself supports present-moment awareness, with several studies observing that participants experienced awe, mental clarity, and expanded perspective in nature settings. Studies that incorporated walking and similar low-impact activities, such as guided forest trails and gentle hiking, reported increased mindfulness, nature connection, and a greater awareness of surroundings (Wolsko and Lindberg, 2013; Nisbet et al., 2019).

Furthermore, NBPA has been shown to facilitate distinct facets of mindfulness, which predominantly contribute to enhanced mental well-being outcomes. A detailed summary of these facets, along with their definitions and associated outcomes, is provided in Table 4.

The types of activities, light to moderate in intensity, allowed participants to become more attuned to their environment without the distraction of physical strain. The findings suggest that environments supportive of mindful immersion are those where individuals can focus on sensory elements of nature, such as sights and sounds while engaging in accessible and non-strenuous NBPAs like easy walking trails and gardening (Kang et al., 2023; Yang et al., 2023).

### 4.3 Is NBPA enhanced because of a deliberate mindfulness intervention?

Evidence from this scoping review varied with the idea that NBPA outcomes can be amplified through mindfulness interventions. For instance, mindfulness techniques added to walking activities enhanced nature experiences by fostering greater awareness, connection, and emotional regulation. Simple mindfulness instructions, such as focusing on the sensory aspects of nature, seem to deepen the benefits of NBPA by encouraging individuals to be still and attuned to their surroundings. This approach offers a promising pathway for practitioners who wish to integrate NBPA with mindfulness, as minimal instructions can support individuals with a gateway to mindfulness without extensive training. Additionally, another study by Schaller and Karing (2024) found no significant additional benefits of combining a mindfulness app usage with nature exposure over a nature exposure or mindfulness app alone intervention. All groups improved in mindfulness, life satisfaction, and stress from pre-to post-intervention and follow-up, but the combined group did not show greater improvements compared to the other groups in any measure. For example, a scoping review that focused on a prolonged nature immersion without any mindfulness practice intervention found participants describing their immersion in nature experiences as fostering a heightened sense of self-awareness, calmness, and creativity, aligning with the concepts of Connectedness with Nature (CWN) and mindfulness (Ward-Smith et al., 2020). These studies support the hypothesis of this scoping review that mindfulness emerges effortlessly in environments supportive of NBPA’s.

### 4.4 How does NBPA facilitate mindfulness to bring about wellbeing outcomes?

Findings across the studies illustrate a link between NBPA, mindfulness, and well-being. Brymer et al. (2020) showed that NBPAs naturally facilitated mindfulness, leading to enhanced mental clarity and presence. Studies such as those by Wolsko and Lindberg (2013) and Ward-Smith et al. (2020) found that participants engaged in activities like hiking, boating, and outdoor education programs reported heightened vitality and mood improvements, directly correlating mindfulness with psychological well-being. Furthermore, the structured walking activities in Nisbet et al. (2019) promoted connectedness, positive mood, and mindfulness, even in the absence of a guided mindfulness practice.

Intriguingly, studies by Yang et al. (2023) and Kang et al. (2023) demonstrated that therapeutic gardening resulted in reduced depression, anxiety and stress, while life satisfaction, daily activities, and mindfulness increased. This suggests that light activities in a green environment such as gardening, are conducive to fostering of mindfulness, particularly in environments such as urban gardens and rural farms.

These findings affirm that the NBPA and mindfulness connection can produce well-being outcomes, as sensory immersion in nature appears to foster positive emotions, stress reduction, and mindfulness.

### 4.5 Interpreting the key findings through the lens of the ED framework

To provide a more conceptual layer of understanding to the key findings, a closer look at the interplay between the person, the environment and the activity (NBPA) as modeled in the ED framework is necessary. Understanding how the results of these studies conceptually make sense in the context of the ED framework, can assist in designing future NBPA interventions that are focused on improving overall wellbeing and health.

The interpretation of the key findings of the studies aligned with the purpose of the ED framework. As shown in Figure 3, presents an adapted model of the ED framework. Each corner of the triangle represents a constraint domain: the individual, the environment, and the activity. The dotted arrows show opposing characteristics, such as low versus high fitness or enabling versus limiting environments. Where these constraints strike a balance (not too challenging, not overly simplistic), the conditions are conducive for the emergence of mindfulness. This aligns with the results of the nine studies as seen in Table 2, ‘Scoping review summary’.

A breakdown of the constraints is necessary to examine how a more quantified and structured approach to NBPA’s can be formulated for future interventions. Importantly, each constraint is defined by two opposing elements, with a dotted marker determining the degree to which that constraint has a lesser or greater predisposition to those particular qualities. This is an important feature to discuss as it offers an insight into how NBPA can be quantified and formulated into interventions accessible to the person and as it relates to that accessibility in certain environments in which NBPAs are performed.

### 4.6 Individual, environment and activity constraints

Within the Ecological Dynamics (ED) framework, constraints refer to the interacting factors within the individual, environment, and the activity that influence behavior. This triadic relationship shapes how nature-based physical activity (NBPA) is experienced and whether mindfulness affordances are likely to emerge.

Individual constraints include a person’s level of fitness, connection to nature, and previous experience with mindfulness. For example, someone new to mindfulness practices or with limited physical capacity may still benefit from NBPA if the task is accessible and immersive.

Environmental constraints refer to the features of the natural setting: its accessibility, level of biodiversity, and whether it’s a curated park or a wilder landscape. These qualities can either support or limit mindful engagement depending on how welcoming and sensory-rich the environment is.

Task (activity) constraints focus on the nature of the NBPA itself: its duration, difficulty, and whether the intention is recreational or competitive. Simpler, low-intensity, and recreational activities (like walking or gardening) tend to offer greater space for mindfulness, particularly when minimal effort is required to engage with the activity.

Rather than viewing these constraints as barriers, the ED framework helps us understand them as dynamic conditions that can be adjusted to facilitate mindfulness. When designed strategically, this person-environment-activity system becomes fertile ground for psychological wellbeing to emerge.

#### 4.6.1 Individual constraints

Individual constraints are made up of (1) nature connection; (2) fitness skill; (3) mindfulness skill.

##### 4.6.1.1 Nature connection (NC)

This takes into account the degree to which a person is connected to the environment while immersing themselves into a complete natural environment devoid of human manufacturing such as paved walkways or trails through a forest. Two variables in measuring adequate NC are eco-centric and anthropocentric constraints. Based on the findings within these studies, a sweet spot for NC is found in the middle of the opposing arrows (see Figure 3). This is important to note, as the findings in the key studies equally focused on the environment and person connection which were in relation to the NBPA undertaken.

##### 4.6.1.2 Fitness skill (FS)

This equates to the individual’s level of fitness, e.g., how cardiovascular fit someone is, while considering the level of skill in the activity they are performing such as an experienced trail walker versus a beginner. The two variables to measure this are low and high levels of FS. The findings in this scoping review suggest a low level of FS, primarily attributed to the minimal fitness and activity demands placed on participants.

##### 4.6.1.3 Mindfulness skill (MS)

This is the degree to which the individual has been trained up or experienced in mindfulness-based practices. The two variables to measure this are low and high levels of MS. The findings in this scoping review suggest a low level of MS primarily due to the low demands of MS required of the participants.

#### 4.6.2 The environment constraints

The Environment constraints are made up of: (1) type, (2) accessibility, (3) requirements.

##### 4.6.2.1 Type

This pertains to whether the biodiversity of the environment is artificially contrived, i.e., parks, or natural such as a forest. Within the studies, most leaned toward natural environments, however it might be argued that forest trails involve human intervention to create an easier or harder trail for example.

##### 4.6.2.2 Accessibility

This equates to whether the environment is easy to conduct NBPA’s or otherwise difficult to perform. Of the five studies, all environment types indicated easy access for the participants to perform the various NBPA’s. It is essential to point out that NBPA can be conducted in urban parks as well as forests that have accessible trails due to human intervention.

##### 4.6.2.3 Requirements

An environment that does not require much in the way of equipment in performing NBPA’s is enabling, while an environment that requires the use of equipment to perform NBPA’s is known as limiting. The results of the nine studies leaned toward environments that were more enabling as equipment was not required in three of the five studies that focused on trail walking only.

#### 4.6.3 The activity constraints

The Activity constraints are made up of: (1) type of task, (2) duration, (3) regulation focus.

##### 4.6.3.1 Type of task

The tasks selected in the nine studies indicated more easy activities such as trail walking. This links closely with the type of environment in which the activities were performed.

##### 4.6.3.2 Duration

Most of the activities were short bouts of NBPA’s (20–90 min). This is consistent with the person type as it relates to the fitness skill requirement being low which correlates with easier and shorter activities.

##### 4.6.3.3 Regulation focus

This concept determines whether a NBPA’s focus is competitive or recreational. The key findings were more focused on the recreational rather than competitive outcomes. This hints that mindfulness affordances might be better induced in a recreational context. While this is not conclusive, examining the nine studies, would suggest this is the case.

In summary, the ED approach is a useful mechanism for understanding how NBPA facilitates mindfulness, specifically highlighting how the person, environment and activity constraints can facilitate mindfulness affordances which lead to wellbeing outcomes (see Figure 3 for a summary).

### 4.7 Summary

This scoping review provides a basis in which a deeper understanding of how wellbeing benefits come about as a result of carefully selected NBPA’s such as moderate trail walks designed to facilitate elements of mindfulness which include a greater sense of perspective and being grounded in the present moment.

With the inclusion of the ecological dynamics’ framework, the discussion of the key findings enriched essential talking points focusing on the inter-relational constraints of person, environment and activity type. For the possibility of mindfulness affordances to emerge, the essential constraints must include the necessary balance in qualities that relate to that activity being easier and accessible rather than difficult, and the skill requirements of both fitness and mindfulness to be less demanding.

The findings from this scoping review carry meaningful implications for those designing wellbeing interventions through nature-based physical activity. Practitioners can draw on these insights to craft experiences that invite mindfulness without requiring formal instruction or training.

First, interventions should aim for simplicity and accessibility. Activities such as short forest walks, therapeutic gardening, or recreational hiking offer gentle entry points into mindfulness states such as present moment awareness. These types of activities are particularly effective when embedded in sensory-rich environments where individuals can tune into the natural environment through seeing, hearing, touching, and smelling while performing a moderate physical activity.

Second, the integration of light mindfulness prompts, like drawing attention to breath, sound, or bodily sensations, can enhance outcomes without complicating the intervention. For participants unfamiliar with mindfulness, this presents a non-threatening, experiential way to cultivate awareness and presence.

Finally, understanding and applying the ED framework allows for interventions to be tailored. By considering the constraints and affordances across the person, the environment, and the task, practitioners can design programs that are responsive to individual needs while leveraging nature’s intrinsic capacity to promote wellbeing.

### 4.8 Limitations of scoping review

While this scoping review has shed light on how NBPA facilitates mindfulness, contributing to wellbeing, it is important to acknowledge certain limitations. Primarily, the limited sample size of studies included in the review restricts the ability to draw definitive conclusions about the nature and extent of this phenomenon. The variation in the methodologies and population types across the studies further complicates the ability to generalize findings.

Additionally, the diversity in the types of NBPAs and environmental settings considered in these studies suggests that NBPA facilitates mindfulness may be context-dependent, varying with different natural environments and activities. The review’s insights are therefore an initial step in understanding this complex interaction.

To build a more comprehensive understanding, future research should focus on expanding the sample size and diversity, including a broader range of NBPA types and population demographics. This will enable a more thorough exploration of how different natural settings and activities can be optimally utilized to enhance mindfulness and wellbeing outcomes.

Developing a more standardized framework, for NBPA such as the ED Framework, including specific guidelines on activity type, frequency, and environmental conditions, would be instrumental in providing tailored recommendations for diverse populations.

Future direction for quantitative research.

While this scoping review lays the groundwork for understanding how NBPA facilitates mindfulness, future research is needed to validate these insights and refine their application. Quantitative studies could explore how different combinations of activity type, environment, and participant characteristics affect the emergence of mindfulness and wellbeing.

For instance, dose–response studies could examine whether more frequent or longer-duration NBPA sessions lead to stronger outcomes. Controlled trials could test whether NBPA with integrated mindfulness prompts yields different results compared to NBPA alone. Researchers might also investigate how various demographic groups respond to different nature settings (urban park vs. wild forest) to better tailor interventions.

Importantly, future work should consider developing ED-informed measurement tools, such as checklists or scales that track individual, environmental, and task constraints, to guide both research and practice. These tools would enhance the capacity to monitor progress and inform best practices for designing accessible, effective interventions.

## 5 Conclusion

Although the sample size is limited, the studies in this review suggest a potential link between (a) NBPA; (b) Mindfulness; and (c) Wellbeing outcomes. This dynamic interplay is yet to be fully understood, however from the nine studies that qualified for this scoping review, the essential findings around increased wellbeing outcomes was clearly demonstrated. This scoping review demonstrated that an opportunity for future studies exists, that are focused around a defined NBPA and mindfulness prescriptive approach for different population types. This review paves the way for innovative approaches such as utilizing the ED Framework in combining NBPA and mindfulness practices for enhanced health and wellbeing outcomes.
